# Organizing for agency: rethinking the conditions for children’s participation in service provision

**DOI:** 10.1080/17482631.2018.1564515

**Published:** 2019-02-01

**Authors:** Anette Bolin

**Affiliations:** Department for Social and Behavioural Studies, University West, Trollhättan, Sweden

**Keywords:** Children’s agency, organizational theory, social work, human service organizations, practice ideologies, practice routines

## Abstract

In the organization of child care services, constraints restrict the potential for children’s participation in the formation and delivery of support programmes. These constraints involve the prioritization of risk management, poor understandings of what participation entails, and entrenched socio-cultural perspectives of children as vulnerable and requiring protection. However, when children’s participation is recognized as an imperative, both morally and as a means of enhancing service efficiency, and when organizational visions and practice ideologies uphold the importance of children’s involvement in decision-making, spaces for children’s agency can become part of everyday practice routines. Drawing on three examples of organizational innovations in child-directed social work, this article explores the benefits involved in “organizing for children’s agency”.

## Children’s participation in social work provision

According to Articles 12 and 13 of the United Nations Convention on the Rights of the Child (UNCRC), children have the right to participate in decisions affecting their lives. For social work professionals, these Articles mean that processes of decision-making in protecting children and supporting their welfare should additionally involve the child (Vis, Strandbu, Holtan, & Thomas, 2011). Children’s participation in decision-making involves the provision of (i) information upon which children can make informed opinions, (ii) opportunities for children to articulate these opinions, and (iii) that professionals take their views into account when designing and delivering care and support (Franklin & Sloper, 2005). However, as noted by Vis and colleagues (Vis et al., 2011; Vis & Thomas, 2009), the implementation of principles concerning children’s participation has proved difficult. The inclusion of children in the making of decisions when structuring programmes of care and support is generally rare, and social workers’ views about the benefits of young service-users’ participation are often ambivalent (Aubrey & Dahl, 2006). Children themselves generally report that they receive little information about support decisions, and feel that they have limited opportunities to participate in decision-making that impacts on their lives (van Bijleveld et. al., 2015; see also Bell, 2002; Bessell, 2011; Leeson, 2007).

Welfare professionals have difficulty recognizing children as agents with capacities to meaningfully engage in their own environments. In a state-of-the-art review, van Bijleveld et al. (2015) investigated the reasons for welfare professionals’ reservations about including children in decision-making. They identify barriers to children’s participation that stem from difficulties in balancing perceptions of the immediate and the longer-term interests of the child. They also identify difficulties in cases when a child’s involvement in care is involuntary. As well as pointing to a general lack of understanding about the nature of participation from a children’s rights perspective, van Bijleveld et al. (2015) also identify constraints restricting the potential for participation that are of an *organizational* nature, and which centre around imperatives relating to risk management. These authors point to entrenched socio-cultural perspectives of children as vulnerable and requiring protection and, from a practice perspective, poor understandings of what participation entails.

As a consequence of these constraints, decision-making about children’s welfare generally takes place in a “procedure-driven, child unfriendly environment” (van Bijleveld et al., 2015, p. 136). Rather than focusing on participation in the narrow context of the child–case worker relationship, van Bijleveld and colleagues suggest that interventions aimed at improving children’s participation need to adopt a wider scope, and should focus on the perceptions of children’s agency and participation that are held by professionals working in welfare organizations. Further, they suggest that “the child should be seen as the service user and the child image should be less focused on protection, and more on that of a child as a knowledgeable social actor.” For these reasons, they argue, children should be afforded “a central position in the decision-making process, right from the start” (p. 137). The review by van Bijleveld and colleagues provides important insights into perceptions of the role of children in decision-making held by social work professionals. However, from an organizational perspective, research is yet to investigate how understandings of children’s agency might influence the structure and forms that provision of support to children in need might take.

Children’s agency is attracting increasing interest in social work research (Chee, Goh, & Kuczynski, 2014; Goh, 2015; Katz, 2015; Praimkumara & Goh, 2015). So far research has focused on documenting and identifying the ways in which children are able to influence decisions that impact on their lives. However, little attention has been paid to children’s actual participation in care processes, or to how opportunities for participation can be supported in the organization of care. Specifically, attention needs to focus on how scope for children’s active participation can be accommodated in the structuring of care provision.

There is a growing awareness that relationships between children and welfare professionals involve bidirectional influences. Just as in families, where parents are more powerful than the child yet are receptive to children’s influence, in professional relationships power is distributed horizontally between clients and service providers (Kuczynski & De Mol, 2015). Interactions between the social worker and the child are now being re-conceptualized as dialogical processes of knowledge exchange (De Mol, Reijmers, Verhofstadt, & Kuczynski, 2018). When interactions between children and case workers are understood as relationally conditioned—i.e., that any interaction can be understood as influenced by the nature of the particular relationship in which it is embedded—opportunities for children’s participation are increased (Goh & Baruch, 2018). These researchers suggest that it is important that professionals take account of children’s knowledge in ways that can enable it to be incorporated into interventions. Further, they argue that when social workers are able to temporarily suspend their professional knowledge in ways that take children’s perspectives into account, this can “open up a space for young persons’ knowledge to be heard” (p. 93). When children’s knowledge is used in this way, the practice validity of support can be enhanced.

## Organizational perspectives on children’s agency

In this article my aim is to scale up the level at which investigation of the role of children’s agency in support provision is carried out. I will argue that there needs to be an understanding of the bidirectional nature of worker–client relationships *at the organizational level*, and that a body of professional knowledge that encompasses recognition of the value of working collaboratively with children needs to be developed. As a consequence, practice can become more systematically oriented towards enhancing children’s participation. When children’s participation is recognized as an imperative both morally and as a means of enhancing service efficiency, and when organizational visions and practice ideologies uphold the importance of children’s involvement in decision-making, spaces for children’s agency can become part of everyday practice routines. In what follows, I critically engage with bidirectional perspectives and conceptualizations of client agency. Then, drawing on examples of organizational innovations in child-directed social work from three separate studies, I explore the benefits of a practice that I have termed “organizing for children’s agency”.

### Human service organizations

Social work is provided by human service organizations (Hasenfeld, 2010a; Hughes & Wearing, 2017). Human service organizations can be distinguished from other service-providing organizations in the nature of the interactions that take place between the users and providers of services. Human service organizations are engaged in moral work, and in upholding values about desirable forms of social behaviour. They gain their legitimacy to work with behaviour-changing interventions both from the wider institutional environment, as well as the dictates of macro-level social policy (Garrow & Hasenfeld, 2010). Efficient service provision is a function of micro-level interactions that take place between the users and the providers of services. As a provider of welfare services, the effectiveness of a human service organization is determined in and through everyday practice. It is for this reason that in the pursuit of efficient service delivery, close connections between the intricacies of everyday interaction, and organizational cultures are required (Hughes & Wearing, 2017).

As a central feature of the work of human service organizations, the agency of service users has received considerable attention. Viewed within an organizational framing, service user agency impacts the service that is produced, and can be defined as “the ability of clients to react and influence the course of the service technology” (Hasenfeld, 2010b, p. 19). In a human service organization, efficient service delivery is the primary objective. However, a similarly designed service provision can differentially affect individual users. As Hasenfeld (2010b) explains, the “reactivity of the clients and their potential capacity to neutralize the effects of the service technology means that the organization cannot take for granted the processes and outcomes of its service technology” (p. 20). Thus, to enhance service efficiency—the likelihood that service delivery takes place as planned—service professionals need to work with clients in order to create conditions under which support can have its intended effects.

### Worker–client relationships and practice ideologies

The relationship between the (service-providing) social worker, and the (service-using) client, constitutes the primary mechanism for achieving beneficial transformations. It is through interactions between workers and clients (both of whom are human agents) that changes in behaviour can be brought about. Worker–client relationships are embedded within sets of assumptions made by service-providers about the people with whom they work. These include implicit evaluations of the moral “worth” of the individual, assumptions about the individual’s own responsibility, assessments of the individual’s amenability to change, and calculations about the value of end results. Most importantly, they involve the view taken of the service user, and whether a client is viewed more as an “object”, or more as a “subject”. Collectively, these value judgements influence the manner in which the service-provider construes the relationship with the service-user:

[W]hether workers treat their clients as objects or subjects determines the extent to which clients will have a voice in what is done to them. Workers who treat their clients as subjects encourage them to become active participants, and to have a voice in decisions about their course of service. In contrast, when clients are treated as objects, they are worked *upon* rather than *with*. (Hasenfeld, 2010c, p. 407).

Worker–client relationships are shaped by organizational forces. There exist a number of mutually recursive influences between the institutional logic of the service-providing organization, and particular relationships between individual users and providers (Hasenfeld, 2010c). At a macro-level, public policy functions to shape the organizational field through which an institutional logic is instantiated. This institutional logic creates an organizational form within which resources are allocated, and through which services are provided. These institutional determinants influence the conditions of work, and shape the nature of relationships.

In human service organizations, organizational routines derive from the particular conditions imposed upon the service provision, and from *practice ideologies* that encompass collective perceptions about the capacity of service-users, their needs, and appropriate responses. Practice ideologies function to reinforce and institutionalize sets of organizational values and moral assumptions, and become embedded in the actions of individual service-providers. They provide both a rationale for discretionary actions, and guidance in carrying them out. Over time, practice ideologies give rise to *practice routines*. These are working routines that shape the patterns of interaction between the people who are providing, and who are using the service. With reference to differences in practice routines, service trajectories can be situated on a relational continuum. At one end of the continuum is bureaucratic processing. Here, service providers relate to their clients as objects, and make decisions that are justified on the basis of manifest facts in relation to existing rules. On the other end of the continuum is professional treatment. Here, service-providers treat their clients as subjects. Decisions tend to be mutually determined, and are “subject to revisions on the basis of active client feedback” (Hasenfeld, 2010c, p 419).

In mapping out the process ecology of work in human service organizations, where levels stretch between enactments of policy, to outcomes for individual service-users, Hasenfeld (2010c) makes clear that the relationships between policies, practice ideologies, and practice routines should not be seen as linear. Rather, processes of top-down and bottom-up interactions operate simultaneously. Equally, causal influences are not unidirectional, but recursive. In this way, while the micro-level, worker–client relationship is conditioned by policy, recursive influences on practice routines and practice ideologies function to modify the moral assumptions of the policies that guide the work carried out:

Frontline workers face conditions of work that require adaptations and improvizations in the face of numerous internal and external resource exigencies. Workers cope by using their own discretion to develop practices that may deviate appreciably from the service logic and technology. Workers rationalize their actions via practice ideologies that, in turn, restructure the service technology in practice. It is the service technology in practice that sets the content and form of worker–client relations and the consequent policy outcomes. (Hasenfeld, 2010c, p. 422)

### A dialectical perspective on practice routines

In Hasenfeld’s model, relationships between levels—i.e., policies, technologies, practices and ideologies—are conceptualized as dialectic (Schmid, 2010). However, a similarly dialectic perspective is not as apparent *within* each of these different levels. Even at the “professional treatment” end of the continuum—where service-user influence is conceptualized to be at its greatest—clients are evaluated in the light of professional expertise. Interactions with clients are valued “as tools to achieve the desired behavioural outcomes”, and workers make use of “persuasion and inducements to achieve compliance” (Hasenfeld, 2010c, p. 420).

In human service organizations involved in the provision of childcare services, a reconceptualization of the worker–client relationship from a dialectical perspective can function in ways that can have an influence throughout the organizational ecology. Changes in the ways that relationships between social workers and their children clients are viewed can take place at any organizational level. As Goh and Baruch (2018) have argued, opportunities for children’s exercise of agency in decision making can emerge through the work of individual social workers, and among smaller groups who share a common belief in the importance of children’s participation. From an organizational vantage point, a dialectical perspective on worker–client relationships of this type can shape the policy of care provision in a bottom-up manner; everyday practice routines of the care-providing organization can develop from exactly the types of practice ideology evident in Goh and Baruch’s (2018) study. My argument is thus that if *at an organizational level* the client–worker relationship is regarded as dialectically constituted, and if this perspective is allowed to inform *organizational innovations*, spaces for children to exercise agency in decision-making can become more systematically characteristic of service provision.

### Innovation: an aligning of organizational visions and practice ideologies

In human service organizations, innovations in the practices of service delivery can have their origins both within, and outside of the organization (Jaskyte, 2010; Jaskyte & Kisieline, 2006; Schmid, 2010). An innovation can result from changes in the wider political, economic, and demographic environment that force the revision of the structural patterns of service provision. Equally, an innovation can emerge from changes in perspectives about the efficacy of service provision within the organization (Schmid, 2010).

In human service organizations, initiatives for change have been shown to arise at all levels of an organization (Cohen, 1999). In in a study of innovation in over 20 non-profit organizations, Light (1998) showed how the origins of innovative change were located at various organizational levels. Some particular types of service-improving change have been categorized as *developmental innovations*. These involve “the modification of existing services for existing users” (Jaskyte, 2010, p. 484). While sources of innovation are often found higher up in an organization’s structure, the legitimacy needed to sustain an innovation often derives from “the professional knowledge and expertise of the people in the system”, and not necessarily the ones “in the top positions” (Jaskyte, 2010, p. 463). Irrespective of whether an innovation has its roots in top-down or bottom-up processes, its influence depends on the extent to which it aligns with professional knowledge and practice ideologies (Hasenfeld & Weaver, 1996).

Recognition of children’s agency and their own knowledge about their own situations, demands that child social work moves beyond the simple solicitation of children’s views as a means of corroborating information provided by adult care providers (Goh & Baruch, 2018). At an organizational level, there needs to be a vision that equates effective child welfare with the active involvement of children. An *organizational vision* of this sort needs in turn to be positively aligned with *practice ideologies* that position children as knowledgeable and competent agents, and with the potential to invest in their own care. This is because it is through practice ideologies—the “shared beliefs about the clients and their needs and appropriate services responses” (Hasenfeld, 2010c, p. 418)—that organizational intentions about the moral importance and contribution to effective service provision of children’s participation can be realized in everyday work. When such an alignment takes place, shifts in perspective about the role of children in shaping care have the potential to be systematic and enduring.

## Organizing for children’s agency: three examples of relationally-oriented practice routines

In the sections that follow, I offer three examples of a practice I have called *organizing for children’s agency*. In each example, I will show how organizational innovations and practice ideologies combine in ways that bring about changes in practice routines in a manner where children’s participation in decision making becomes a resource in service provision. In each case, policy-generated organizational innovations designed to increase service efficiency demanded that social work professionals worked in collaboration with those in other welfare fields to produce more effective forms of support. In this inter-professional climate, and in the interests of effective service delivery, practice ideologies emphasizing collaboration between professionals, and between workers and clients, resulted in practice routines incorporating active measures for facilitating children’s participation.^1^

### Practice routines involving accessibility

In the first example the principle of “organizing for children’s agency” was accomplished by making support possibilities more visible, and more easily accessible.

The case in focus here comes from a study I conducted with Emma Sorbring (Bolin & Sorbring, 2017). Over the period of a year, we carried out ethnographic research at a school where a larger than average proportion of students came from socially and economically disadvantaged backgrounds, and where a team of social workers provided on-site child and family support services. Services were directed at both individual and group problems. They included family group meetings, contacts with individual students, social assessment investigations, and “open-door” counselling. On a group level, team members carried out long-term focused interventions to address problems at the school, such as bullying and alcohol use. For individual students, these programmes focused, for example, on skills development and impulsive behaviour control, while a series of evidenced-based programmes designed to develop/increase parenting skills were provided for parents. In addition to the on-site provision of welfare services, teachers at the school were provided with in-classroom support by the social workers, along with various forms of counselling and guidance in addressing the behavioural problems of students in their classrooms.

In Sweden, it is normal practice for social workers to be based at schools, and to offer counselling services. However, at this school the extent and comprehensiveness of the service provision was highly unusual. The programme was initiated as a consequence of a complex web of interrelated problems involving parenting, economic disadvantage, disruptive behaviour and low educational achievement. When designing the innovation, one of the primary aims was to overcome service-users’ generally sceptical responses to social support, and the reluctance of many families in need to seek help from service-providers because of perceived stigma. It was for these reasons, that it was decided to place the social worker team at the school. Overall, the aim of the intervention was to encourage service-use, and to increase pupils’ self-referrals through extending opportunities to access support from service-providers who, in this organizational form, had a highly visible presence in the everyday school environment.

Tasked with increasing support to a wider client base, a work model was developed where support was neither sourced nor provided through traditional channels. This was achieved by the social workers making themselves more accessible to pupils and offering support when pupils themselves sought help. This approach differed significantly from traditional practice. There were no fixed referral mechanisms such as, for example, traditional case-allocation meetings or processes where social workers would take on a case referred to them by another department or agency. Although it emerged that the social workers had very clear ideas about which children needed support, they were not directly proactive in establishing contact with these children. Rather, contacts were established through the organizational technology of enhancing accessibility.

The social workers facilitated contacts by making themselves accessible to children’s initiations. For example, their office was located in one of the school corridors, meaning that the team members became familiar faces in the school environment. The social workers participated in various curricular and extra-curricular activities. They also established an online presence, using social media as a medium through which contact could be initiated and maintained, even outside school hours.

Because they were required to work in an unusual setting, and with colleagues who possessed different forms of expertise, the social workers were provided with the opportunity to re-conceptualize their professional knowledge (Goh & Baruch, 2018):

Because we are working in a school setting, then I think that we get to see the whole spectrum [of the children’s problems] and in another way. Like, I think that you get more perspectives on what it is that we want to achieve. [School social worker]

As a response to environmental conditions where contacts with social services were not positively viewed, either by pupils or their parents, the social workers developed new practice routines. These routines were underpinned by a practice ideology where the children themselves should be enabled to become the initiators of contact, and the instigators of support:

We made it so that we were available, so that they could get in contact. It makes things easier for us if they get in contact themselves. They become change-oriented. … We have confidence in the children’s resources and capacities. They can gain more power if they come [to us] when they have already defined the problem. They also come earlier, and the problem may not have become too overwhelming. And so it is easier to bring about change. [School social worker]

The model created a relational hierarchy radically different to that normally pertaining between social workers and clients. This was achieved by dismantling many of the traditional structures and practices in service provision. Instead of regulated but infrequent encounters that took place on a site that was far away from the school (and which was negatively perceived and rarely voluntarily visited), support was provided in the everyday contexts of the students’ school routines, and through social media channels. This model was also very different to what these children had previously experienced. The organizational technologies of increasing visibility and accessibility, and changes in practice routines involving contact-initiation, enabled the children to act instrumentally in the making choices on matters with an impact on their lives. Because children actively sought out support on their own initiation in a relationship where vertical structures of power were less pronounced than normal, the children were able to make greater self-investment in care provision.

### Practice routines involving talk

In the second innovation involving the principle of “organizing for children’s agency”, social workers who were collaborating with teaching staff at a resource school created dedicated spaces for therapeutic talk in pupils’ timetables. The case comes from an ethnographic study of co-located inter-professional collaboration where social workers and teachers worked together in providing educational and social support (Bolin, 2011). Here, the organization of work at the school involved innovative forms of professional practice as solutions designed to address the needs of children in families with complex social and educational problems. In addition to working in ways defined by their profession-specific functions, as teachers and social workers, the staff also worked interchangeably in delivering services. For the social workers, an important profession-specific function involved the carrying out of structured therapeutic talk.

In the practice routines developed by these social workers, talk sessions with the pupils were scheduled into their weekly timetable, in the same way as the subject lessons. Because the pupils’ school-related problems were multi-dimensional, and were interlinked with problems relating to their family situation, some of these scheduled sessions were also held together with the pupil’s parent(s). The therapeutic talk was part of the pupils’ schedule. Not only did this mean that it was symbolically elevated to the same status as key academic subjects, but in each session the children and their parents were tasked with setting agenda.

Like many of the families in the previous example, these pupils and their parents had generally negative experiences of contacts with welfare agencies. Here, too, an innovative practice ideology was developed that involved recognition of the knowledge and resources possessed by pupils and their parents. The goal was to increase client investment by enabling children and their parents to become involved in the co-construction of behaviour-changing programmes of support, and by utilizing their expert knowledge of their own situation. This practice ideology is articulated in views about the agency of the children and their parents:

Quite simply, if we cannot get the parents and the children on board, and make use of their resources, there will not be any change. We might as well just disband the whole project. [Social Worker]

Because the structured therapeutic talk was specifically designed to increase investment, it was decided that it should take place in the room with the most inviting environment. Unlike the layout and furnishings of the school’s classrooms, and meeting-rooms generally found in schools and the offices of municipal welfare-providers, this room was modelled on a family living room. The lack of an institutional architecture can be understood as more readily facilitating client investment. The “living room” environment signalled a departure from the vertical power-structures normally governing interactions between case-workers and clients, and which are symbolically represented in the institutional furnishing of social work offices. As a practice routine, holding talk in this living-room setting was intended to enhance client participation.

The talk was structured in ways where, in advance of each occasion, the students and their parents were invited to identify topics around which discussion would centre. As in the previous example, this practice routine enabled clients to become the initiators of directions that the talk would take. Specifically, the more horizontal distribution of power provided greater scope for the clients’ agency and participation. It enabled the children and their parents to become more instrumental in the design and planning of support. It involved a shift from a more deterministic logic that privileged professional knowledge and sought client compliance, to a logic more relational in nature, where solutions were jointly worked out. This meant that support provision became organized around opportunities and solutions that the service-users were themselves involved in identifying:

It [the talk] should be the pupil’s time. The opportunity to talk about things. In principle it can be anything. It doesn’t have to be difficult stuff, and can just as well be something that functions well./…/We have for example a girl who has experienced some pretty horrible things, so then we talk about these things. But it much depends on the pupil herself, at that particular time. If there is something special that they want to talk about [Social Worker]

### Practice routines involving jointly created care packages

In the third example, the principle of “organizing for children’s agency” was accomplished by co-locating multiple services on a single site, and in a way that enabled young service-users to become more actively involved in the construction of packages of care (Bolin, 2016). Child care services that had previously been provided by different agencies were relocated to a specially initiated, multidisciplinary centre, “The Family House”, in order to improve service provision. The centre housed seven different services, and was in a building entirely separate from other municipal offices. These were: a family (marital) guidance team, a family support team (providing services to parents), a social network team, a drug treatment team, a detached youth work team, a youth placement prevention team, and a youth intervention team, the latter tasked with providing interventions based on multi-professional assessments.

Because it brought together teams working with young people and families in a single organizational structure, the “Family House” setup was unusual in a Swedish context. The innovation of co-locating and coordinating child and family welfare services had two explicit aims. The first was to create an organizational structure for interdisciplinary service provision which could improve service efficiency through holistic solutions. The second aim was to increase young people’s participation in their own care by enabling them to interact with a smaller number of specially selected key workers, and by reducing the number of offices that they were required to visit.

In different policy texts, including mission statements and annual audits, it was made explicit that care interventions at the “Family House” should be designed to take place within the young person’s own social network, and that institutional or foster care should be avoided. These policies were embraced by the professionals working at the “Family House”, and were enacted within day-to-day practice routines that involved working in ways that encouraged the children and young persons to be active in decision-making. Importantly, these policies were shaped by the belief that the success of an often complex, multi-party intervention was dependent on the young person’s own participation:

It is central that the child is involved in the intervention and its design. Because a child is a part of the system. There an equally important cog in the machine, like all of the others. Each voice is equally important. It would almost feel like an impossibility if we were not to involve the children in things. It is more like, why would *not* involve them? It is possible to work without participation, but we think this is wrong. Most of all it is lacking in respect. You just ignore the child and decide things over their heads, as if they were stupid. [Social Worker]

The message about holistic service-provision and service-user participation was made visible and discussed with the clients. This took place not only at the outset of care packages, but also during the trajectory of the service-provision. This can be understood as an evolving practice routine. Three different spaces for participation were identified in the practice routines at the “Family House”: involvement in the identification and evaluation of alternative service options, involvement in the creation of the forms and structures of provision, and involvement in evaluation processes. The children and young people were actively encouraged to participate in decision-making. This meant that they gained opportunities to become involved in co-constructing care-packages and, as in both of the previous examples, to become invested in their own care as part of a process of personal development.

Spending time on site with these children, I found that they experienced having scope to exercise their agency. This was indicated by comments about how their wishes were taken seriously, even in situations where desires or objections could cause problems. When they reflected on the scope available for participation, the young people talked about ways in which they had intentionally influenced the nature and forms of the support they were given, and how and when support was provided. They spoke about the effects that such influence had on their investment in relations with individual social workers, and in the overall care process. In the following excerpt from an interview with one young person, the space for agency created by this flexible and holistic organizational approach is clearly illustrated:

I: OK, if we talk about this thing about how you can be involved and have influence power and control, when you are with Karin [case-worker], exactly how do you feel that you can influence things?

R: I can influence quite a lot, I think. We often decide things together. We make a schedule together. And should there be something I don’t want to do, then usually we don’t do it. So I feel really that I can [influence things] and that she gives me lots of alternatives. Like she doesn’t just make a choice, but always asks what I think. Stuff like that.

I: And how often, because you have been meeting for a year and a half now, roughly how often do you meet?

R: Roughly 2–3 times a week.

I: Have you been able to decide when you meet?

R: Yes.

I: Has it been that you have not wanted to meet, like ‘I don’t want to meet for a couple of months’?

R: I have never actually felt like that, but I feel certain that I could have said that, but I would probably have regretted it after a while.

I: So you have wanted to meet her and done so and decided each time, pretty much trouble-free?

R: No, well, there were some times when I felt pretty low and haven’t had the energy to meet anybody, but on those times I have said so, and she has understood.

I: Sure. So 2–3 times a week you meet Karin and do things…

R: We go out with her dogs, or my dog, and sometimes we go for walks, or paint pictures, or we go somewhere and film a bit, sometimes just talk, sometimes go for a coffee.

For this young person, the recognition that she is able to actively influence and shape the provision of care through interactions that she perceives to take place on her own terms, means that she is positively disposed to involvement. Within a worker–client relationship where decision-making power appears as more horizontally aligned, she is able to exercise her *relational agency* (De Mol et al., 2018). As a consequence, she becomes invested in a process where support is co-created.

## Organizing for children’s agency: the creation of zones of discomfort

In the theories of human service organizations that currently dominate the conceptualization of service provision in child-focused social work, client compliance is a major concern in the management of the service trajectory (Hasenfeld, 2010b). Service-user agency is framed as a “technological indeterminacy”, and a potential obstacle standing in the path of an efficient service trajectory. Client agency therefore requires control. The service user–service provider relationship is the means by which the unpredictability and uncontrollability of service-user agency can be controlled, and through which compliance can be achieved. In this sense, theories of human service organizations share common ground with branches of child psychology where, by tradition, children’s attempts to achieve autonomy and express preferences are framed using clinical concepts such as “noncompliance” (Kuczynski & De Mol, 2015).

Kuczynski and De Mol (2015) take issue with the idea that positive clinical outcomes can be achieved through processes of control, and via measures designed to limit noncompliance. They refer to work by Wilson (2007, 2012) who describes family therapeutic practices in which children are included as important actors. Wilson’s argument is that children’s agency, and the creativity and unpredictability that it brings, should not be understood as a threat or a problem. Rather, it should be seen as a resource. Like adults, children too should be viewed as agents of change. For service-providing professionals, acknowledgement of the generative potential of children’s agency can mean that they need to learn to tolerate a zone of discomfort in their dealings with clients. This is a zone where they may not know what to do, or how to control the client’s behaviour.

For the social workers in the examples previously referred to, unpredictability was not so much a “price worth paying” in encouraging participation, but rather a necessary part of being genuine in taking children’s agency seriously by supporting the children’s own initiatives. It meant working almost permanently within a zone of discomfort:

Thinking purely in the short term, it is perhaps easier to work without participation. We, and the parents, would have got what we wanted. The child would be compliant in the short term. But this would be situationally secured, and would not be good in the long-term. Instead, we would get a child who would be vulnerable, and with poor well-being. … Of course it [working with participation] demands much more time, but it pays off in the long-run, because we get a better result if we think in terms of them [the children] being participants. [Social Worker]

In the work with families and children described in these three examples, zones of discomfort can perhaps best be understood as *ambiguous spaces* within professional practice that are *knowingly* and *deliberately* entered. Although characterized by uncertainty, the work conducted in these spaces is recognized as providing insights into problem areas from the child’s perspective, and opportunities for development that might not otherwise have existed. Working in ways that take explicit account of children’s agency, and in spaces characterized by unpredictability, means that social workers need to adopt an “active attitude” (Kuczynski & De Mol, 2015, p. 355). It requires the recognition that, because outcomes are co-constructed within the worker–client relationship, they cannot be rigidly predetermined.

In closely parallel theorizing, Hasenfeld (2010c) explains how, as a consequence of shifts in practice ideologies, a “zone of discretion” can materialize within practice routines where moral assumptions may differ from those more generally affirmed within a particular institutional logic. The scope of such a space, Hasenfeld (2010c) argues, will be dependent on any sanctions that might place limits on discretion. However, because most people-changing polices lack strict enforcement capabilities, in the delivery of welfare provision, zones for discretion can be potentially quite wide.

As I have framed it here, in the context of child-centred social work, a “zone of discomfort” is situationally emergent. It arises in particular relational and organizational conditions, and it evolves through strategic choices that are made in the creation of innovative organizational solutions. Through the practice ideologies that arise in social workers’ engagement with organizational visions in daily practice routines, children and young people may become able to participate in the creation and formation of care as knowledgeable and agentic individuals. In the three examples described here, the development of innovative, inter-professional approaches to working with young people and families with complex needs was informed by a recognition that their investment in service provision could be enhanced if they were able to engage as active participants. In each case, a traditionally structured service-delivery operating under a mechanistic–deterministic organizational logic was transformed into a multi-disciplinary undertaking that was client-responsive, and which operated under a dialectical–relational logic. In relation to the service-user children and their families, causal mechanisms of control and compliance were supplanted by relationally-oriented visions of participation coupled with recognition of a need to work within zones of discomfort. As I have described in relation to these examples, because predictability and compliance were replaced by flexibility, accommodation and negotiation, the young people were able to invest in a package of care from the position of being a co-constructor.

## Conclusion

In this article, I have argued that when creation of space for children’s agency becomes an organizational technology, and a part of everyday practice routines, the relational resources and transformational capacity of the worker–client relationship can be productively used in developing care that is effective as a consequence of increased investment on the part of the child or young person. As a parameter in the design of care, “organizing for children’s agency” can take place at all levels of practice, from the structuring of individual meetings and case conferences, to the organization of service provision at institutional levels. This position is consistent with the argument of van Bijleveld et al. (2015) that children need to be seen as service users who are knowledgeable social actors, and should from the outset be afforded a central position in the decision-making process. In order to ensure that children are indeed engaged as knowledgeable participants, service providers should consistently ensure that children are involved in all stages of the care process. This, I argue, demands a reconceptualization of what is meant by effective service provision. It demands that deterministic conceptualizations that position children as the “objects” of care provision, and worker–client relationships as the “tools” through which compliance is assured, give way to dialectical conceptualizations, where children are seen as *active* agents, where agency is viewed as *relational*, and where relationships become *transactional* accomplishments.

The practice of social workers in different settings is shaped by cultural, institutional and contextual factors. While the organizational innovations drawn on in this article as examples of “organizing for children’s agency” show how changes in institutional policy and the structuring of service provision can influence children’s involvement in decision-making, it needs to be recalled that child social work in Sweden is generally informed by the perspective that children should be listened to, and should be involved as active participants in decisions affecting them (Rasmusson, Hyvönen, Nygren, & Khoo, 2010). In other settings, where the practice of child social work is not child-centred to the same degree, and where perceptions of the role of children in society may be less accommodating, the structuring of care in forms where spaces for children’s active participation are created needs to be carried out in parallel with efforts directed to bringing about changes in societal, institutional, and practice ideologies.

## Note

1. The studies referred to in this article were all carried out following the appropriate codes of research ethics. In accordance with the Ethical Review Act, ethical approval was sought and obtained from the Regional Ethical Review Board in Gothenburg, Sweden for Bolin and Sorbring (2017) (reference number 718–13), and Bolin (2016) (reference number 029/12). In both of these studies information about the study and the ethical requirements was given orally and in writing. Informed consent, verbally and in writing, was obtained from the child participants, their parents (for those children 15 under), and the staff at the respective organizations. In Bolin (2011), where focus was on the professional practice of adult members of staff, approval from the Regional Review Board was deemed unnecessary, and no application was made. In this study, the staff were informed of the purpose of the study and the relevant ethical requirements orally and in writing, and consent was obtained orally and in writing.
